# BDNF as a Biomarker of Cognition in Schizophrenia/Psychosis: An Updated Review

**DOI:** 10.3389/fpsyt.2021.662407

**Published:** 2021-06-16

**Authors:** Rodrigo R. Nieto, Andrea Carrasco, Sebastian Corral, Rolando Castillo, Pablo A. Gaspar, M. Leonor Bustamante, Hernan Silva

**Affiliations:** ^1^Departamento de Psiquiatría y Salud Mental, Facultad de Medicina, Universidad de Chile, Santiago, Chile; ^2^Clínica Psiquiátrica Universitaria, Hospital Clínico de la Universidad de Chile, Universidad de Chile, Santiago, Chile; ^3^Departamento de Neurociencias, Facultad de Medicina, Universidad de Chile, Santiago, Chile; ^4^Facultad de Psicología, Universidad San Sebastián, Santiago, Chile; ^5^Departamento de Neurología y Psiquiatría, Clínica Alemana Universidad del Desarrollo, Santiago, Chile; ^6^Millennium Nucleus to Improve the Mental Health of Adolescents and Youths, Universidad de Chile, Santiago, Chile; ^7^Biomedical Neuroscience Institute, Universidad de Chile, Santiago, Chile; ^8^Programa de Genética Humana, Instituto de Ciencias Biomédicas, Facultad de Medicina, Universidad de Chile, Santiago, Chile

**Keywords:** brain-derived neurotrophic factor, neurotrophin, neurocognition, cognitive symptoms, chronic schizophrenia, first episode psychosis, clinical high-risk for psychosis

## Abstract

Brain Derived Neurotrophic Factor (BDNF) has been linked to cognitive symptoms of schizophrenia, which has been documented in previous reviews by several authors. However, a trend has recently emerged in this field moving from studying schizophrenia as a disease to studying psychosis as a group. This review article focuses on recent BDNF studies in relation to cognition in human subjects during different stages of the psychotic process, including subjects at high risk of developing psychosis, patients at their first episode of psychosis, and patients with chronic schizophrenia. We aim to provide an update of BDNF as a biomarker of cognitive function on human subjects with schizophrenia or earlier stages of psychosis, covering new trends, controversies, current research gaps, and suggest potential future developments in the field. We found that most of current research regarding BDNF and cognitive symptoms in psychosis is done around schizophrenia as a disease. Therefore, it is necessary to expand the study of the relationship between BDNF and cognitive symptoms to psychotic illnesses of different stages and origins.

## Introduction

Brain Derived Neurotrophic Factor (BDNF) is the most widely distributed neurotrophin in the brain and it has been associated with several psychiatric disorders, including schizophrenia and other psychotic disorders. The relationship between BDNF and cognitive symptoms in patients with schizophrenia has been described in previous reviews by several authors (1–5), including one from our group (6).

The search for molecular biomarkers of cognition has been an important field of research in patients with schizophrenia, in which BDNF has been identified as one of the main ones, along with others such as inflammatory markers (2). The molecular pathways involved in schizophrenia form complex and interconnected networks, where BDNF is one of the points where different risk factors converge (7). It should be noted that BDNF has been investigated as a diagnostic or prognostic biomarker in various psychiatric pathologies, including schizophrenia, but also depression, bipolar disorder, as well as anxiety disorders. It has been related in these pathologies with different symptomatic domains, including the cognitive, but also the emotional and social domains, so it would not be a pathognomonic or specific marker of any particular disorder or symptomatic group (8).

In the literature there are studies on BDNF both in animal models and in human beings, including healthy subjects and patients with any of the mentioned diagnoses. Methods to approach the study of BDNF in patients include measuring the level of this protein in plasma or blood serum (9) or studying variations at the genetic level. Some single nucleotide polymorphisms (SNP), such the Val66Met polymorphism, have been associated both with decreased neurocognitive function in healthy adults and with clinical aspects of various psychiatric disorders. Research on this polymorphism has increased significantly in the last decade, and several studies have related it to the pathophysiology of schizophrenia, psychotic symptomatology, cognition, efficacy and side-effects of antipsychotic drugs (3, 10). Considering that recently some research projects are no longer studying categorial diagnoses such as schizophrenia, but syndromes such as psychosis (11), it is important to update the review of the relationship between BDNF and cognition in psychotic patients.

In this narrative review we aim to provide an update of BDNF as a biomarker of cognition on human subjects during different stages of the psychotic process, covering new trends, controversies, current research gaps, and suggest potential future developments in the field.

## Methods

We performed a search in the Medline/PubMed database with the terms BDNF, schizophrenia and cognitive/cognition. We then repeated the search replacing the word schizophrenia with psychosis. A total of 280 articles were found in the first case (schizophrenia), while 87 in the second (psychosis). With the aim of updating our previous review published in 2013 without repeating what had been already reported, we selected articles that were published during the last seven years (2013–2020, including those available only as Epub in 2020). Therefore, we had two lists of 237 and 64 articles that were selected for further review, although many of the “psychosis” articles were already included in the “schizophrenia” articles list. Among all of these, we selected articles on BDNF and cognitive function in human subjects with psychosis/schizophrenia that contained information of *BDNF* gene (such as Val66Met polymorphism) or BDNF peripheral levels (plasma or serum). No standardized approach was used since our aim was to produce a narrative review. We only included articles that were available in English. Animal studies were excluded in order to keep the focus and length of this mini review article within limits.

In order to organize our findings, recent BDNF studies in human subjects were classified into one of three different stages of the psychotic process: subjects at high risk of developing psychosis, patients at their first episode of psychosis, and patients with chronic schizophrenia. These are the following subsections that readers will find in this article. Since the latter has more information available, the chronic schizophrenia subsection has been further divided into pharmacological and non-pharmacological interventions.

## In Subjects At High Risk of Developing Psychosis

We found three recent studies that investigated the relationship between peripheral blood levels of BDNF and cognition in subjects at risk of developing psychosis. One of these groups (12) carried out a cross-sectional study in patients who were in one of three possible stages of the disease, including “at-risk mental state” (ARMS). They found higher BDNF levels in patients with chronic schizophrenia, intermediate levels in patients with a first psychotic episode, and lower levels in ARMS. These findings suggest a possible drop in BDNF levels prior to the onset of psychosis. In this study, plasma BDNF levels were related to some cognitive functions in all groups such as the ability to plan.

Sanada et al. (13) studied another population of subjects considered at risk of developing psychosis, according to the criteria of “ultra-high risk” (UHR), and found lower BDNF levels in this UHR group in comparison to a group of healthy control subjects. They found no relationship between plasma BDNF levels and the cognitive domains studied, neither in UHR nor in the group of healthy subjects. After a period of follow-up, they observed that BDNF levels in UHR subjects persisted at similar levels for at least 6 months (13).

Consistently, He et al. (14) reported lower levels of plasma BDNF in a group of “clinical high-risk” (CHR) subjects, in this case in comparison to patients with first episode psychosis. In this study, the CHR subjects had lower levels of both mature BDNF (mBDNF) and precursor BDNF (proBDNF), as well as lower levels of receptors in BDNF pathways: TrkB and p75NTR. They also found a weak negative relationship between TrkB levels and scores in the Stroop Color-Word Test.

It should be mentioned that in subjects who have not developed a psychotic episode, but who present schizotypal personality traits, it has been observed that the Val66Met polymorphism of *BDNF* can affect learning ability. In the study by Skilleter et al. (15), participants with the *BDNF* Met allele and with higher schizotypal traits showed poorer learning ability.

## In Patients With First Episode Psychosis

Recently, publications on the relationship of BDNF and cognition in patients with first episode psychosis (FEP) have focused on drug-naïve patients. Xiao et al. (16) studied a group of 58 patients without exposure to antipsychotics and a control group, finding that the patients had poorer cognitive performance in almost all the neurocognitive tests they used. However, BDNF levels were inversely correlated with some tests (TMT part B), and positively with others (VTF-action). It is worth mentioning that these findings stood in the same direction when studying the correlations of these cognitive tests with the levels of another neurotrophin (“Glial cell line - derived neurotrophic factor,” GDNF).

Man et al. (17) recruited a group of 80 patients with FEP and no exposure to antipsychotic treatment. They reported lower BDNF levels in patients compared to control group subjects, as well as a lower neurocognitive performance in patients in almost all the cognitive domains studied (except the index of visuospatial ability). They found no relationship between BDNF levels and the results of cognitive evaluations. However, they reported that the negative subscale of the PANSS (Positive and Negative Syndrome Scale) was negatively correlated with the indices of immediate memory and language. In accordance with this, the Val66Met polymorphism of *BDNF* has also been related to the clinical characteristics of the patients (predominance of positive or negative symptoms) in a genetic study in treatment-naïve patients (18).

Recently, Wu et al. (19) investigated the effect of risperidone monotherapy on cognitive impairment in drug-naïve first-episode of psychosis patients (DNFE) and whether BDNF levels were correlated to improvement of cognition. They found that lower BDNF levels were correlated with delayed memory in DNFE patients with high baseline BDNF levels. After 12 weeks of treatment, risperidone significantly improved immediate memory, delayed memory and RBANS total scores and BDNF levels were slightly increased. In patients with low baseline BDNF, BDNF levels were significantly increased after risperidone treatment, while in patients with high baseline BDNF, BDNF levels were significantly decreased. In addition, baseline BDNF levels were associated with improvement of delayed memory and were a prognostic factor for the improvement of delayed memory and RBANS total score in patients with high baseline BDNF levels.

Xiu et al. (20) suggested that interrelationships between BDNF and antioxidant mechanisms might underlie the pathological mechanisms of cognitive impairments and symptomatology in DNFE patients with schizophrenia. In their study, DNFE patients had reduced levels of BDNF compared with healthy controls, and BDNF levels were positively correlated with copper/zinc-containing superoxide dismutase (SOD) activity in patients. Moreover, an interactive effect of BDNF levels and manganese-SOD activity was associated with attentional index score in the patients. In a different study, Qu et al. (21) found that the manganese-SOD was significantly correlated with attention, language and RBANS total scores, but only in male patients. Multiple linear regression analysis showed that the interaction between BDNF and manganese-SOD was associated with RBANS language index score in male patients. Previously, Wei et al. (22) showed that an interaction between BDNF and oxidative damage is involved in the disruption of executive function in patients with chronic schizophrenia.

Other studies in patients with FEP that have failed to find an association between BDNF and cognitive symptoms, have instead found an association between BDNF and negative symptoms. This is the case of the study by Mezquida et al. (23), which found a relationship between the Val66Met polymorphism of *BDNF* and the severity of negative symptoms. Additionally, low BDNF has been related to depressive symptoms in schizophrenia (24).

Some studies on FEP patients that also looked for and found no relationship with cognitive symptoms, found a relationship with a history of abuse in childhood. Theleritis et al. (25) found a significant effect of a history of separation, physical abuse, and sexual abuse on BDNF levels, with lower levels in those who experienced the traumatic event compared to those who did not. These findings suggest a possible role for BDNF in the onset of psychosis in individuals exposed to early trauma, which proposes it as a potential biomarker of the deleterious effects of childhood trauma on brain plasticity (25). Additive effects between childhood trauma and *BDNF* Val66Met polymorphism Met carriers have been found on volume loss of the hippocampal subregions cornu ammonis (CA)4/dentate gyrus and CA2/3 in schizophrenia patients (26). This might be related to the methylation of BDNF gene in association with childhood maltreatment reported in schizophrenia patients by Barker et al. (27).

Other authors, such as Yang et al. (28), have recruited both FEP patients and chronic schizophrenia patients in their study. They found significant correlations between BDNF levels and partial cognitive dimensions such as visual learning, memory and processing speed.

## In Patients With Chronic Schizophrenia

In patients with chronic schizophrenia, cognitive deficits have been related to various factors, including the severity of psychotic symptoms, age, medication, as well as BDNF levels. Atake et al. (29) studied the associations between these variables in a group of patients, observing that the levels of BDNF in serum were correlated with working memory, attention, processing speed, motor function, and executive function. A recent meta-analysis of BDNF levels and cognitive impairment in schizophrenia (30), that included 21 studies that accounted for a total 2,449 patients with schizophrenia-spectrum disorders, found that BDNF levels were modestly but significantly related to cognitive functioning. Considering cognitive domains, BDNF levels were significantly associated with verbal memory, working memory, processing speed and verbal fluency performances.

Ahmed et al. (31) had previously performed two separate meta-analysis, considering both *BDNF* gene and levels. In the first one they studied the association between the Val66Met polymorphism and neurocognition in people with schizophrenia, and found non-significant difference between the genotype groups on most neurocognitive domains. In the second one they studied the association between cognition and peripheral expression of BDNF, and found minimal yet significant correlations for the reasoning and problem-solving domains.

A key neural circuit for cognitive control, such as the connectivity between the anterior cingular cortex (ACC) and prefrontal regions (PFC), has been found to be influenced by the Val66Met polymorphism (32), suggesting a possible mechanism connecting cognitive symptoms to genetic and environmental risk factors of schizophrenia. Regarding the study of relationships between genetic polymorphisms of the *BDNF* gene and cognitive alterations in patients with schizophrenia, it should be noted that this has been done not only with the Val66Met polymorphism (also known as rs6265 or G196A), but also with other polymorphisms (with positive results for rs12273539 and rs10835210). The latter showed a significant effect specifically at the language level (33).

Male patients with schizophrenia, compared to female patients, have shown worse results on some tests of immediate and delayed memory. Accordingly, these male patients have shown lower levels of BDNF than these female patients. A positive relationship of BDNF with immediate memory and with the total score of the RBANS cognitive battery has been observed in female patients, but not in men (34). It should be noted that, in the same study, in the healthy control group no differences were observed between men and women in the cognitive outcomes, nor in BDNF levels, neither in the BDNF/cognition relationship. Studies that have delved into the relationship between the Val66Met polymorphism of *BDNF* and cognition separating groups of men and women have also found gender-specific associations. In female patients with the Val allele, shorter reaction times and fewer correct responses were observed both in the continuous performance test (CPT) and in the trail making test (TMT parts A and B). However, these associations between Val allele and cognition were not evident in male patients (35).

Patients with an earlier age of onset have shown more negative symptoms and cognitive deficits, as well as lower levels of serum BDNF. In these patients, negative symptoms partially mediated the relationship between age of onset and cognitive deficits, which was moderated by serum BDNF and Val66Met polymorphism, since the mediating effect of negative symptoms exhibited a Met allele dose-dependent tendency (36).

In a study carried out in patients with paranoid subtype schizophrenia, it was observed that the majority of these patients showed some degree of cognitive impairment, but with considerable variability between different individuals. Among these patients, those with lower concentrations of BDNF had lower processing speed (37). Tang et al. (38) studied a group of patients with deficit schizophrenia and found that BDNF levels were lower in those patients in comparison to non-deficit schizophrenia, although not significantly.

In patients with chronic schizophrenia who have presented tardive dyskinesia (TD) during their evolution, lower levels of BDNF have been observed than in patients without this complication. Likewise, in patients with TD, worse results have been found in various cognitive evaluations, including immediate memory. Interestingly, BDNF levels correlate negatively with immediate memory in patients with TD, but positively in patients without this complication, which may imply different underlying neurobiological mechanisms (39).

Li et al. (40) studied the relationship between serum BDNF levels and cognitive dysfunction in schizophrenia patients comorbid with type 2 diabetes mellitus (T2DM). They found that schizophrenia patients with T2DM had significantly higher serum BDNF levels than schizophrenia patients without T2DM. Also, schizophrenia patients with T2DM scored higher in delayed memory than schizophrenia patients without T2DM. The authors suggested that the increase of BDNF levels and better cognitive performance, particularly delayed memory, may be related to the pathophysiological process of T2DM in chronic schizophrenia patients.

### In Relation to Pharmacological Treatment

In patients diagnosed with schizophrenia in monotherapy with an atypical antipsychotic, positive correlations have been observed between BDNF levels in serum and scores for verbal memory, attention, and processing speed (41). The same group, in a separate publication, addresses patients with schizophrenia specifically on aripiprazole treatment, finding an association between BDNF levels and the cognitive domains of memory and verbal learning, verbal fluency, and executive function (42).

Regarding the treatment of patients with schizophrenia who suffer an acute relapse, Zhang et al. (43) prospectively studied the evolution of a group of patients treated with olanzapine. During the decompensation of their disease, these patients had worse cognitive results than a control group, as well as lower BDNF levels. After a 12-week treatment period, the patients improved in terms of positive and negative symptoms (according to PANSS), improved some cognitive parameters (immediate memory, attention, and total score of the RBANS cognitive battery), and had an increase in levels plasma levels of BDNF. Correlation analyzes showed that this increase in BDNF levels was correlated with the change in the cognitive score described.

Koola et al. (44) proposed that the galantamine-memantine combination may concurrently improve BDNF, oxidative stress, antioxidant, and anti-inflammatory biomarkers. Considering the interaction between BDNF and oxidative stress in schizophrenia, and the association of oxidative stress with positive, negative and cognitive symptoms, they suggested that antipsychotic-galantamine-memantine combination may provide a novel strategy in schizophrenia to treat positive, negative and cognitive symptoms.

In recent decades there has been growing concern about the metabolic effects of atypical antipsychotics, which may have an impact on cognitive dysfunction. It has been observed that those patients with chronic schizophrenia on long-term treatment with olanzapine and who suffer metabolic syndrome have lower scores in both immediate and delayed memory, attention, and total score in the RBANS battery, as well as lower levels of BDNF (45).

More recently, Tang et al. (46) hypothesized that omega-3 fatty acids may be of value in enhancing BDNF levels and improving cognitive function in patients with schizophrenia with metabolic syndrome (MetS). They recruited 80 patients with both schizophrenia and MetS who received long-term olanzapine monotherapy, and found a significant correlation between omega-3 fatty acid treatment and enhanced delayed memory factor in the RBANS assessment when the patients completed this study. They found that along with cognitive improvement, omega-3 fatty acids enhanced BDNF levels in these patients. Guo et al. (47) showed that omega-3 fatty acids improve cognitive impairments through Ser133 phosphorylation of CREB, upregulating BDNF/TrkB signal in patients with schizophrenia.

### In Relation to Non-pharmacological Treatments

Several studies have investigated the relationship between non-pharmacological interventions and peripheral BDNF levels in patients with schizophrenia. A recent meta-analysis, which included both initiatives based on cognitive training and others based on physical exercise, found that BDNF levels were increased in patients participating in these types of non-pharmacological interventions (48).

Regarding the effect of cognitive rehabilitation, Fisher et al. (49) showed that participants in computerized auditory training had significant improvements in global cognition, processing speed, learning, and verbal memory. These patients had lower than normal BDNF levels before starting training, that increased significantly during training to reach normal levels post-intervention (compared to a control group). Penadés et al. (50) also showed that patients with schizophrenia who participated in a neurocognitive rehabilitation study had significant improvements in cognition and quality of life, but did not detect significant changes in BDNF levels in the whole group. They related this finding to genetic variants of the Val66Met polymorphism of *BDNF*, observing that the Val/Val patients had significant increases in BDNF levels in relation to this intervention. Serum BDNF has been suggested as a predictor of response to neuroplasticity-based cognitive training in a recent review by Biagianti et al. (51).

Studies of galvanic skin response biofeedback training in schizophrenia patients have also found a statistically significant increase in BDNF serum levels after a 3-month follow-up period (52). A study protocol has been reported for measuring BDNF levels in schizophrenia patients undergoing yoga therapy, but results have not yet been published (53).

Regarding the effects of physical exercise, several studies have shown both an improvement in the performance of various cognitive tests and significant increases in BDNF levels. Many of these studies have found a positive correlation between this increase in BDNF and cognitive improvement, suggesting a molecular mechanism for the clinical effect. However, some studies have not observed this relationship, so it might be still premature to draw definitive conclusions about this issue (54, 55). While aerobic exercise related cognitive benefits have been linked to BDNF upregulation, this putative mechanism needs confirmation (56). Holmen et al. (57) found an association between cardiorespiratory fitness and cognition in schizophrenia patients, but they did not find evidence of BDNF mediating this association. The study with the largest sample to date (58) found that BDNF mRNA was positively associated with both physical activity and cognitive functioning. A smaller but more recent study by Massa et al. (59) found that an increase in BDNF levels after 12 weeks of aerobic training was associated with a significant increase in reasoning and problem solving.

Some authors have suggested to combine the effects of physical exercise and cognitive training to obtain better results (60, 61), based on the promise that each of these strategies has individually represented for the improvement of cognitive deficits in patients with schizophrenia. Starting aerobic exercise in close temporal proximity with cognitive remediation strategies may allow the brain to better achieve a state prone to neuroplasticity, facilitating the improvement of neurocognitive functions. Li et al. (62) suggested coupling physical exercise with dietary glucose supplement for treating cognitive impairment in schizophrenia. There is not enough clinical evidence yet, only pilot studies, but future studies are expected to incorporate these promising strategies. As Jahshan et al. (63) have explained, given that cognitive remediation may depend on intact neuroplasticity to produce cognitive gains, it is reasonable to combine it with strategies that harness patients' neuroplastic potential.

## Discussion

The role of BDNF as a biomarker of different symptomatic domains in several psychiatric pathologies has been studied from various points of view. Its relationship with cognitive dysfunction in patients with different stages of psychosis, including but not limited to chronic schizophrenia, accumulates growing evidence. This review article has focused on recent studies of the relation between cognition and both *BDNF* gene polymorphisms and BDNF peripheral levels, in human subjects during different stages of the psychotic process, including subjects at high risk of developing psychosis, patients in their first episode of psychosis, and patients with chronic schizophrenia. Our findings are summarized in Table 1.

**Table 1:** BDNF and cognition in human subjects during different stages of the psychotic process.

**Group of patients**	**Specific group**	**Main findings**	**References**
Subjects at risk of developing psychosis	ARMS	Lower BDNF levels in subjects at risk of psychosis BDNF levels related to cognition, ability to plan	(12)
	UHR	Lower BDNF levels in subjects at risk of psychosis BDNF levels not related to cognitive tests	(13)
	CHR	Lower BDNF levels in subjects at risk of psychosis TrkB levels related to SCWT scores	(14)
Patients with first episode psychosis (FEP)	Drug Naïve FEP	Lower cognitive performance in FEP than controls BDNF levels inversely related to some cognitive tests BDNF levels positively related to other cognitive tests	(16)
	Drug Naïve FEP	Lower cognitive performance in FEP than controls BDNF levels not related to cognitive tests	(17)
	Drug Naïve FEP	BDNF levels and Mn-SOD related with attentional index	(20)
	Drug Naïve FEP	BDNF levels and Mn-SOD related to language index	(21)
	Risperidone treatment	Baseline BDNF levels associated with improvement of delayed memory and RBANS total score	(19)
Patients with chronic schizophrenia	Meta-analysis	BDNF levels related to verbal memory, working memory, processing speed and verbal fluency	(30)
	Meta-analysis	*BDNF* levels related to reasoning and problem solving BDNF Val66Met related to most neurocognitive domains	(31)
	Chronic schizophrenia	BDNF levels related to working memory, attention, processing speed, motor and executive function	(29)
	Chronic schizophrenia	*BDNF* Val66Met related to ACC-PFC connectivity	(32)
	Chronic schizophrenia	*BDNF* rs10835210 related to language index	(33)
	Female patients with chronic schizophrenia	BDNF levels related to immediate memory and RBANS total score. Higher score on immediate and delayed memory, and higher BDNF levels, compared to men.	(34)
	Female patients with chronic schizophrenia	*BDNF* Val allele related to shorter reaction times and fewer correct responses in CPT and TMT A and B	(35)
	Patients with earlier age of onset	*BDNF* levels and BDNF Val66Met moderate mediating effects of negative symptoms between age of onset and cognitive deficits	(36)
	Paranoid schizophrenia	BDNF levels related to processing speed	(37)
	Deficit schizophrenia	BDNF levels lower in deficit schizophrenia patients than in non-deficit schizophrenia patients	(38)
	Schizophrenia and Tardive dyskinesia	Lower BDNF levels and lower scores in cognitive evaluations in TD patients. BDNF levels correlate negatively with immediate memory in TD patients	(39)
	Schizophrenia and diabetes mellitus 2	Higher BDNF levels and delayed memory scores	(40)
In relation to pharmacolgical treatment	Atypical antipsychotic treatment	BDNF levels related with verbal memory, attention and processing speed	(41)
	Aripiprazole treatment	BDNF levels related to memory and verbal learning, verbal fluency and executive function	(42)
	Olanzapine treatment	Increase of BDNF levels during relapse treatment related to improvement in cognitive parameters	(43)
	Olanzapine treatment and metabolic syndrome	Lower levels of BDNF and lower levels of immediate and delayed memory, attention and RBANS total score	(45)
	Omega-3 fatty acids treatment	Increase of BDNF levels and improvement in delayed memory	(46)
	Omega-3 fatty acids treatment	Upregulation of BDNF/TrkB signal and improvement of cognitive impairments	(47)
In relation to non-pharmacological interventions	Meta-analysis	Increase of BDNF levels in patients that participated in non-pharmacological interventions	(48)
	Cognitive remediation	Increase of BDNF levels and improvements in global cognition, processing speed, learning and verbal memory	(49)
	Cognitive remediation	Improvements in cognition and quality of life Increase of BDNF levels only in Val/Val patients	(2)
	Physical exercise	Increase in BDNF levels, improvement of cognitive tests BDNF increase may be related to cognitive improvement	(54) (55)
	Physical exercise	BDNF mRNA related to both physical activity and cognitive functioning	(58)
	Physical exercise	Increase in BDNF levels related to improvement in reasoning and problem solving	(59)

The identification of biomarkers that are clinically useful to psychiatric disorders is one of the most challenging tasks in psychiatric research and of major concern in modern medicine (64). Since cognition is one of the most important predictors of community functioning in schizophrenia (65), the search for biomarkers that can predict or relate to cognitive function in patients with psychosis is a clinically significant field of research. Peripheral BDNF levels may be particularly useful as a biomarker of cognition in schizophrenia because levels in plasma or serum can easily be measured from a blood sample, and may change during different stages of the illness, or in response to pharmacological and non-pharmacological interventions. According to Pan et al. (66), intact BDNF in the peripheral circulation can cross the blood-brain barrier by a high-capacity, saturable transport system. Pillai et al. (67) reported a significant positive correlation between plasma and cerebrospinal fluid (CSF) BDNF levels in FEP subjects. They concluded that parallel changes in BDNF levels in plasma and CSF indicate that plasma BDNF levels reflect the brain changes in BDNF levels in schizophrenia. All of this suggests that changes in peripheral BDNF relate to changes in CNS functions in schizophrenia, having an impact on cognition as shown in this review.

Pharmacogenomics is a promising field for a better understanding of both side effects and response to antipsychotics (68). The *BDNF* Val66Met variants could be a promising target for choosing antipsychotic medication options or for developing next generation antipsychotic drugs. However, further studies will be required in this area to confirm the effect of this polymorphism in patients' response to antipsychotic drugs, including patients from different ethnic populations (10). According to this review, *BDNF* Val66Met could be further studied as a predictor of clinical improvement of cognitive symptoms in response to antipsychotics or to a combination of antipsychotics with other pharmacological and non-pharmacological treatments.

Despite awareness of the need for early interventions, fewer studies on BDNF and cognition are found in patients with a first psychotic episode than in patients with established chronic schizophrenia. It should be noted that publications on BDNF and cognition in subjects considered at high risk of developing psychosis have begun to appear, but these studies are still scarce and include a relatively small number of subjects. Considering that finding a reliable biomarker for the early detection of schizophrenia has been a topic of interest over the last decade (69), this seems to be a promising potential subject matter for future research.

On the other hand, the novel tendency represented by the RDoC model to be able to study biomarkers in patients with psychosis as a group, instead of using the traditional diagnostic categories of the DSM or the ICD (11), does not seem to have permeated the field of study of BDNF and cognitive symptoms. Still, most of the research regarding BDNF and cognitive symptoms in psychosis is carried out in relation to schizophrenia as a disease, so implementing studies devoid of the bias of traditional diagnostic categories represents a future challenge for this line of research.

It is worth mentioning that the groups of patients studied have been expanded, including patients with no exposure to antipsychotic treatment, patients classified according to subgroups (e.g., paranoid schizophrenia), or patients grouped according to side effects of medications (e.g., patients with tardive dyskinesias). Regarding cognitive tests, only a few studies use consensus initiative batteries currently validated such as MATRICS (70) or CNTRICS (71) which represents a limitation to compare different studies. An important part of the articles described in this review have studied not only the association between BDNF and cognitive symptoms, but also that with other symptomatic domains, highlighting the finding of its association with negative and depressive symptoms.

Our review has several limitations to consider, including searching only in the Medline/PubMed database and including only articles in English language. However, most high impact research is published in this format. Additional limitations are that we wrote a narrative review instead of a systematic review, and that we focused only on recent articles (2013 and onwards). Finally, not including studies in animal models limits the possibility to enhance discussion with translational research. Despite being beyond the focus of our review, studies in animal models of schizophrenia and pre-clinical studies with post-mortem brain tissue in humans are an active field of BDNF research that can be found in other review articles (72, 73).

It seems necessary to extend the study of the relationship between BDNF and cognitive symptoms to psychotic conditions of different stages and origins. Longitudinal studies and longer follow-up periods will be useful to better understand BDNF variations during the evolution of the psychotic process. BDNF may be a useful biomarker in psychosis for early diagnosis, although the question about whether it can be helpful to predict conversion to schizophrenia in CHR subjects remains open. In patients with FEP or chronic schizophrenia, future pharmacological and non-pharmacological studies should consider what we know about BDNF, particularly as a biomarker of cognition, in order to guide potential therapeutic strategies for the improvement of cognitive symptoms.

## Author Contributions

All authors listed have made a substantial, direct and intellectual contribution to the work, and approved it for publication.

## Conflict of Interest

The authors declare that the research was conducted in the absence of any commercial or financial relationships that could be construed as a potential conflict of interest.
